# Supplementary material for: Progression of type 1 diabetes from latency to symptomatic disease is predicted by distinct autoimmune trajectories
Source: Nat Commun. 2022 Mar 21;13:1514. doi: 10.1038/s41467-022-28909-1 (PMC8938551; doi:10.1038/s41467-022-28909-1)
Supplement: Supplementary file 1 — Supplementary Information [file 41467_2022_28909_MOESM1_ESM.pdf]

Supplementary Information

Manuscript Title: Progression of Type 1 Diabetes from Latency to Symptomatic Disease is Predicted by Distinct Autoimmune Trajectories

Authors: Bum Chul Kwon[1#*], Vibha Anand[1#], Peter Achenbach[2], Jessica L. Dunne[3], William Hagopian[4], Jianying Hu[5], Eileen Koski[5], Åke Lernmark[6], Markus Lundgren[6], Kenney Ng[1], Jorma Toppari[7], Riitta Veijola[8], Brigitte I. Frohnert[9], the T1DI Study Group[&]

Affiliation: 1. IBM Research, Cambridge, Massachusetts, United States; 2. Institute of Diabetes Research, Helmholtz Zentrum München, German Research Center for Environmental Health, Munich-Neuherberg, Germany; 3. JDRF, New York, New York, United States; 4. Pacific Northwest Research Institute, Seattle, Washington, United States; 5. Center for Computational Health, IBM Research, Yorktown Heights, New York, United States; 6. Department of Clinical Sciences Malmö, Lund University CRC, Skåne University Hospital, Malmö, Sweden; 7. Institute of Biomedicine and Centre for Population Health Research, University of Turku, and Department of Pediatrics, Turku University Hospital, Turku, Finland; 8. University of Oulu and Oulu University Hospital, Department of Pediatrics, PEDEGO Research Unit, Oulu, Finland; 9. University of Colorado, Denver, Colorado, United States

[&]All main authors are members of the T1DI Study Group. A list of authors and their affiliations appears at the end of the paper.

[#]**Equally contributing authors**

[*]**Corresponding author:** Bum Chul Kwon, bumchul.kwon@us.ibm.com

Supplementary Figures

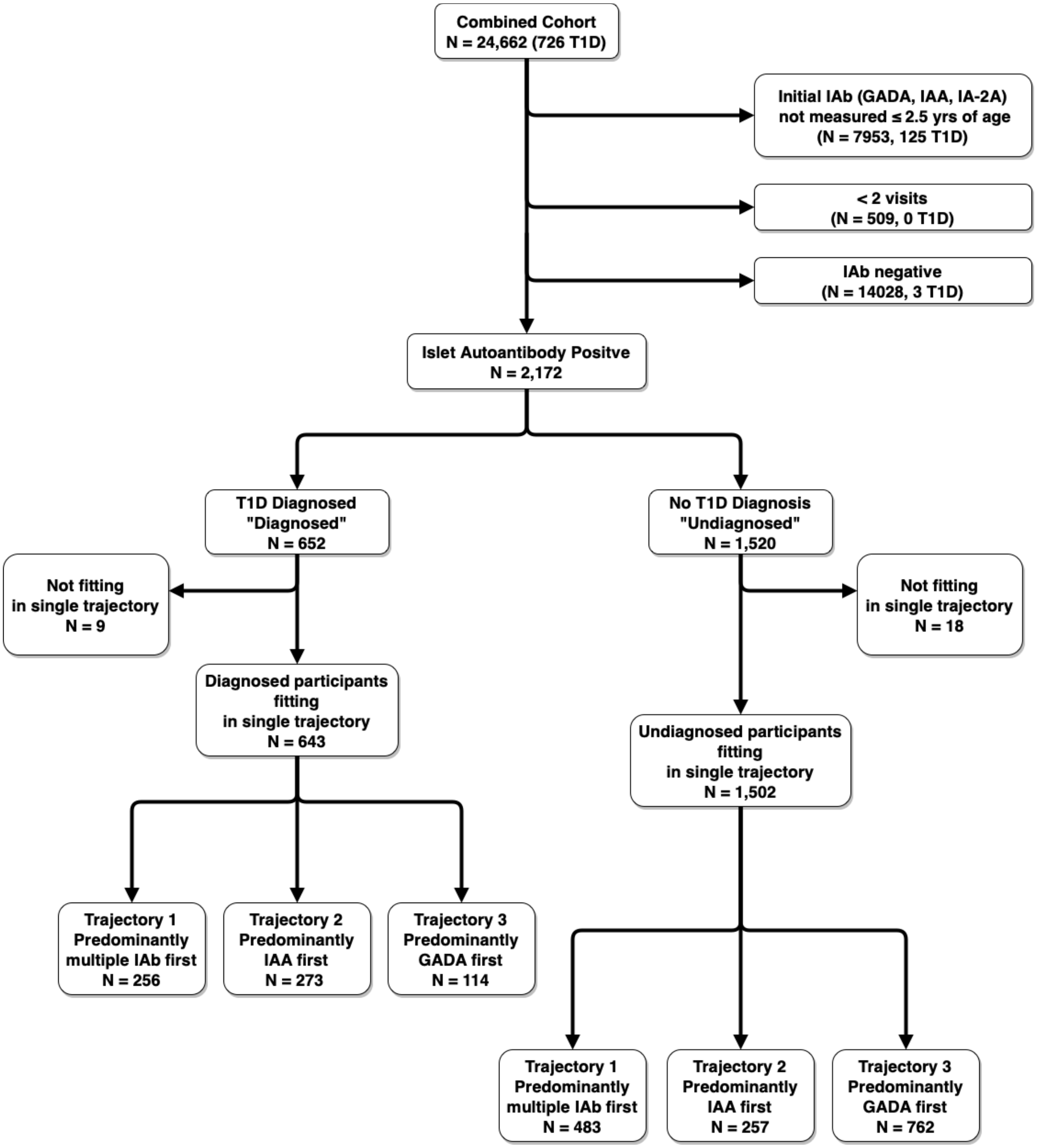

Supplementary Figure 1. Flow chart of participants analyzed in the manuscript. *The flow chart shows the cohorts we used in the manuscript. Of 24,662 participants recruited from the five prospective studies, 2,172 participants were selected by the following procedure: 1) we removed 7,953 participants (125 participants with T1D diagnosed at the end) who were not measured on initial Islet Autoantibodies (IAb), namely GADA, IAA, and IA-2A,earlier than 2.55*

years of age; 2) Then, we removed additional 509 participants (0 T1D) who were only measured once because we wanted longitudinal data for disease progression modeling; 3) Finally, we also removed 14,028 participants (3 T1D) who did not show a positivity in any IAb through their observations. 2,172 participants can be divided into 652 participants who were diagnosed (D) with T1D during study follow-up and 1520 participants who were not (UD). We applied the learned Hidden Markov Model to fit the participants into the 11-state model. We found that each of 2145 (643 T1D) participants (98.8%) fit into one of three trajectories; the remaining 27 participants (9 T1D), who did not fit in a single trajectory, were pulled from the analysis. Diagnosed participants can be categorized into TR1: 256, TR2: 273, and TR3: 114 participants; Undiagnosed participants can be categorized into TR1: 483, TR2: 257, and TR3: 762 participants.

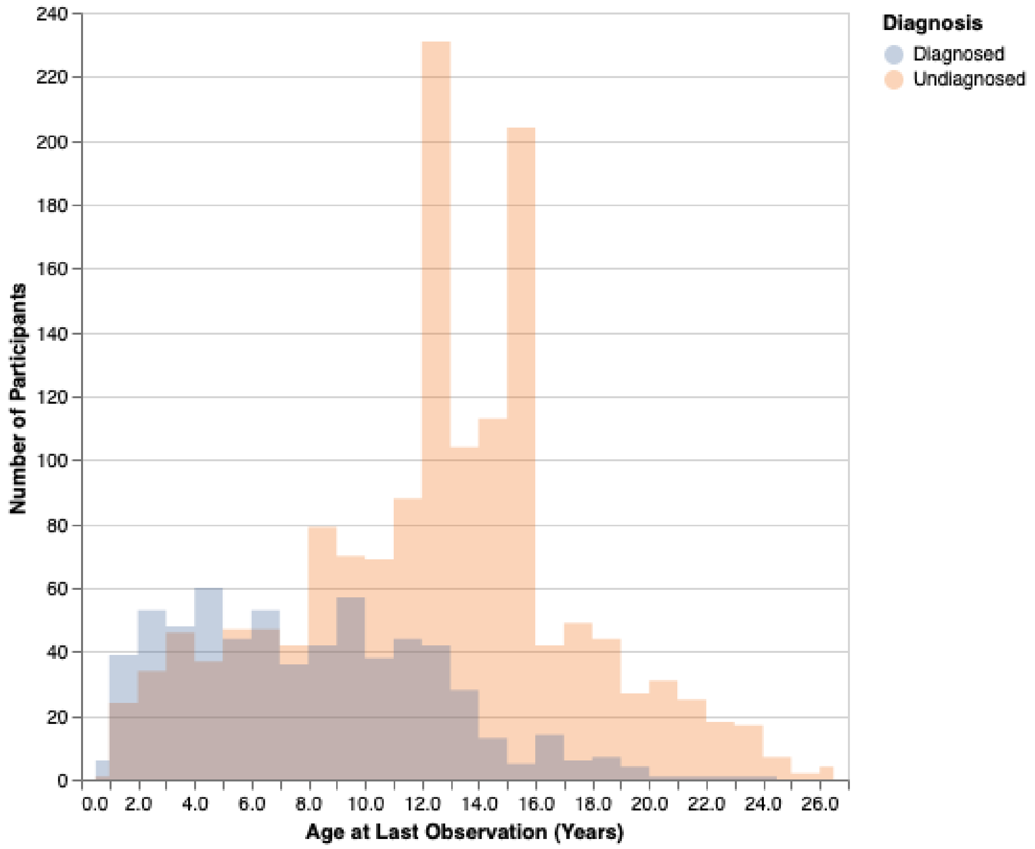

Supplementary Figure 2. Histogram of participants' age at their last observations. The histogram illustrates participants' age at their last observations of the diagnosed (N=643) and the undiagnosed (N=1502) participants. The median age of the diagnosed participants' last observation, which represents the age at diagnosis, is 7.62 years old (IQR, 4.19 to 11.22). On the other hand, the median age of the undiagnosed participants' last observation is 12.87 years old (IQR, 9.29 to 15.42).

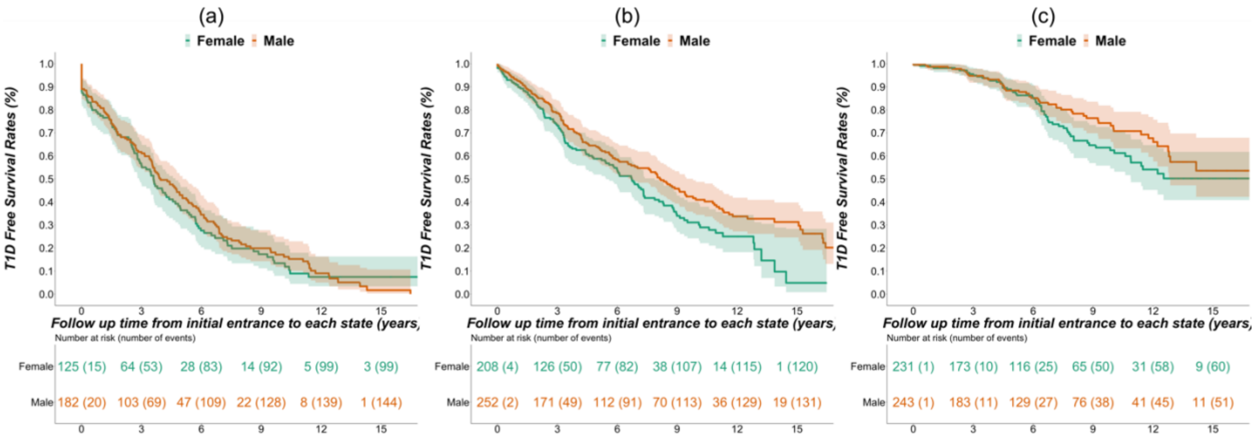

Supplementary Figure 3. Diabetes-free survival rates stratified by sex for each trajectory. T1D free survival rates (mean and 95% confidence interval) of female and male children in TR1 (Panel A), TR2 (Panel B), and TR3 (Panel C). There is a significant difference between male and female children in TR2 (Log-rank, $Z^2(1) = 5.7$; $p = .02$). There is no significant difference between male and female children in TR1 (Log-rank, $Z^2(1) = 0.3$; $p = .6$) or in TR3 (Log-rank, $Z^2(1) = 2.3$; $p = 0.1$).

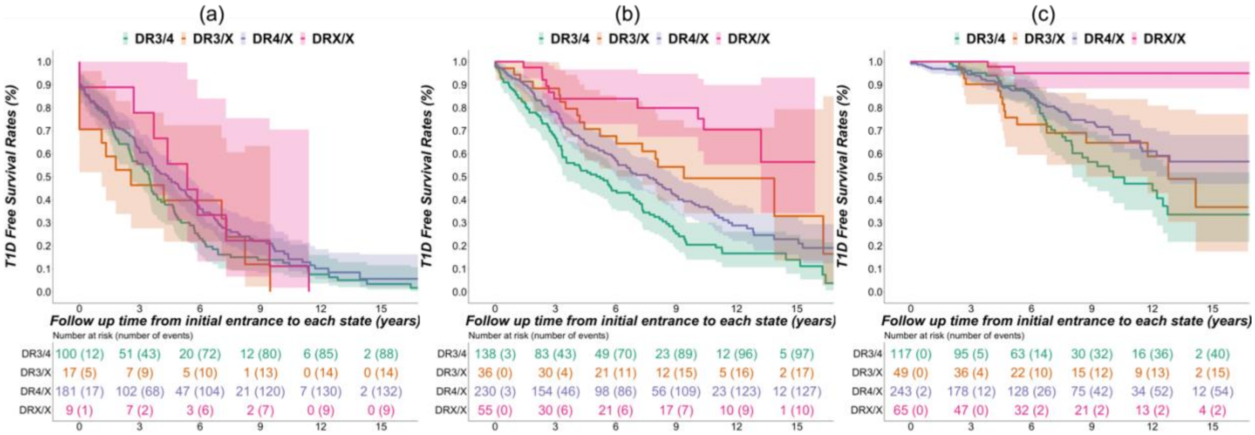

Supplementary Figure 4. Diabetes-free survival rates stratified by HLA for each trajectory. T1D free survival rates (mean and 95% confidence interval) of children with different HLA-DR status in TR1 (Panel A), TR2 (Panel B), and TR3 (Panel C). There is no significant difference among children with different HLA-DR status in TR1 (Log-rank, $Z^2(3) = 3.7$; $p = 0.3$). There are significant differences among children with different HLA-DR status in TR2 (Log-rank, Z2(3) = 31.6; $p < 0.0001$). The pairwise comparison test shows significant differences in following pairs: i) DR3/4 and DR3/X ($p = 0.003$); ii) DR3/4 and DR4/X ($p = 0.003$); iii) DR3/4 and DRX/X ($p < 0.0001$); iv) DR4/X and DRX/X ($p = 0.001$). There are significant differences among children with different HLA-DR status in TR3 (Log-rank, $Z^2(3) = 19.7$; $p < 0.0001$). The pairwise comparison test shows significant differences in following pairs: i) DR3/4 and DR4/X ($p = 0.0041$); ii) DR3/4 and DRX/X ($p < 0.0001$); iii) DR3/X and DRX/X ($p = 0.0006$); iv) DR4/X and DRX/X ($p = 0.0017$).

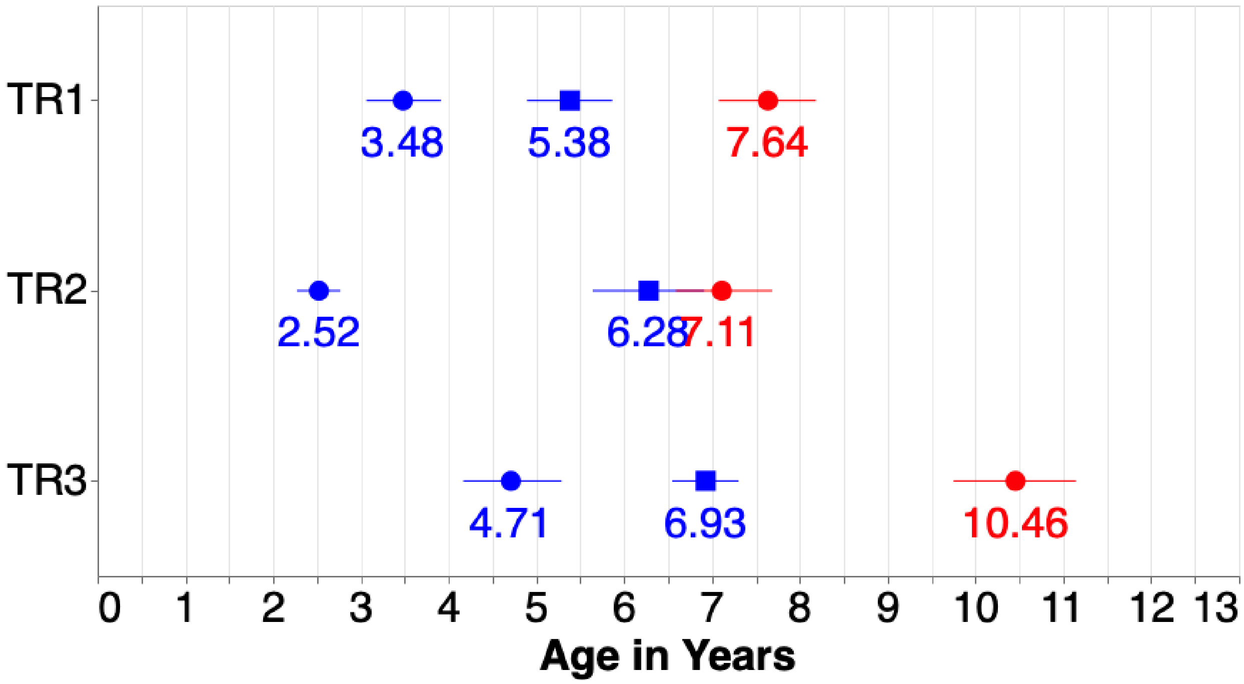

Supplementary Figure 5. Age at seroconversion and clinical onset for the three trajectories. Mean and 95% confidence intervals of age at confirmed seroconversion and clinical onset of diabetes. Mean and 95% confidence intervals of age at confirmed seroconversion (blue) for the diagnosed (circles) participants (N = 546) and the undiagnosed (squares) participants (N = 840) and clinical onset (red) of the diagnosed participants (N = 643) between four each of three trajectories.

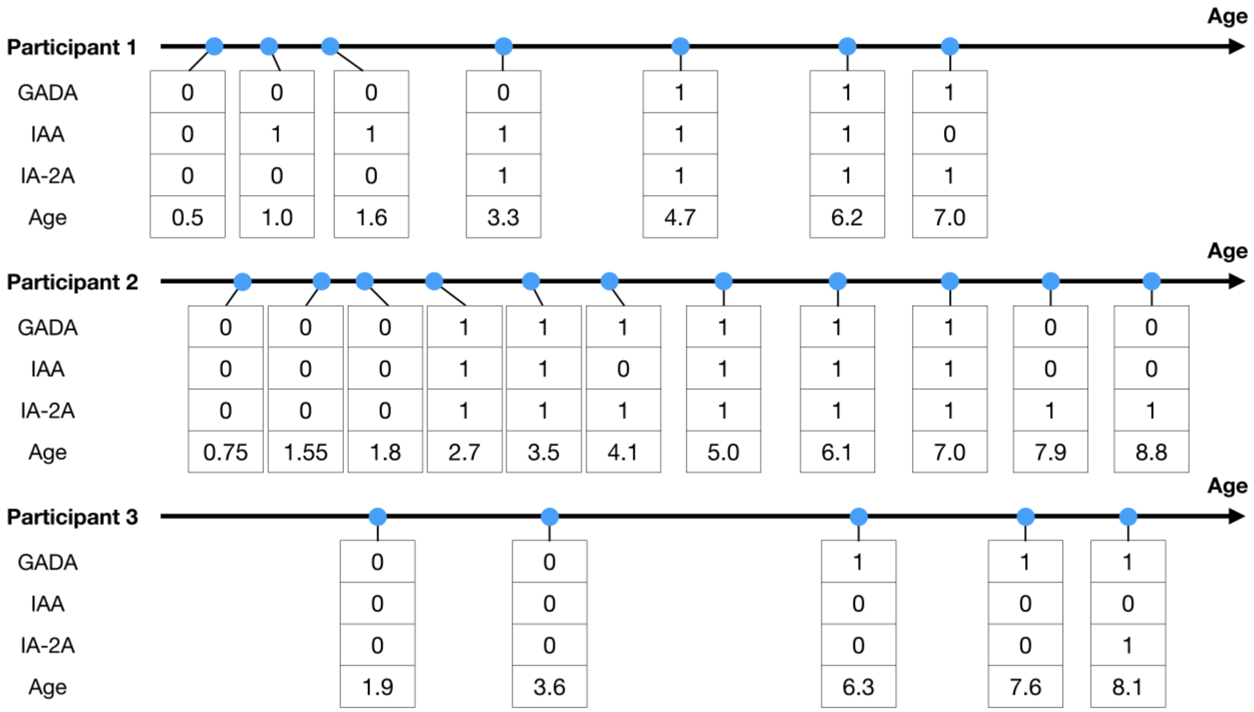

Supplementary Figure 6. A graphical illustration of the input data structure. The diagram illustrates the input data structure for the Continuous-Time Hidden Markov Models (CT-HMM) we used in the study. Participants have varying numbers of visits over their age with irregular time intervals and each visit includes three tuples of islet autoantibodies, namely GADA, IAA, and IA-2A, and age as their measurements per visit.

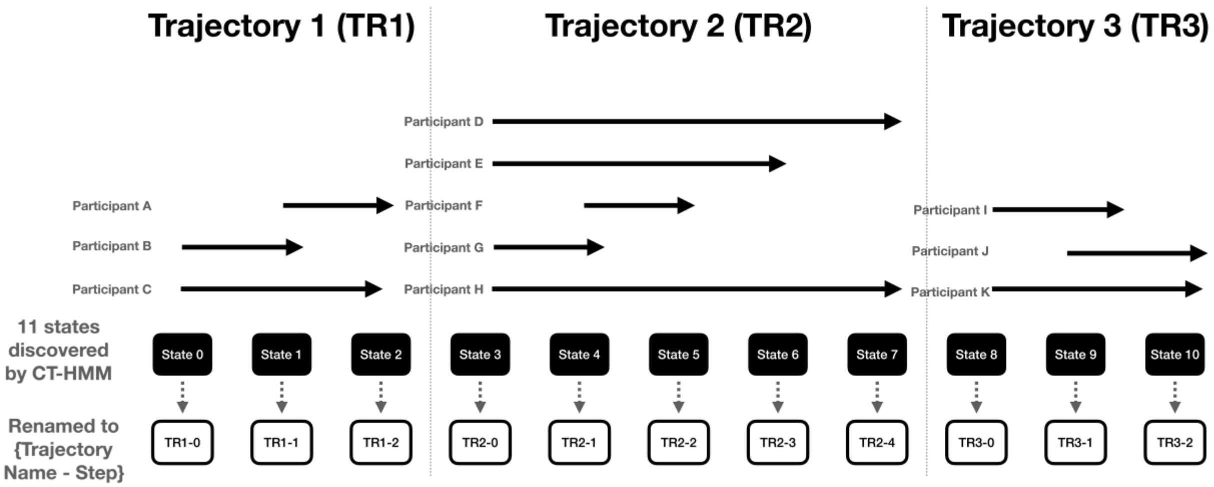

Supplementary Figure 7. A graphical illustration of the 11 states described in the manuscript. Inspecting the 11-state model identified by CT-HMM and applied to the analysis cohort, we observed that each participant started and ended their observations within one and the same trajectory out of the three trajectories: Trajectory 1 consists of three states, Trajectory 2 includes five states, and Trajectory 3 has three states. To more clearly describe this the distinct patterns of the three trajectories in the manuscript, we renamed the 11 states to the {Trajectory Name –Step within Each Trajectory} format, e.g., TR1-1.

Supplementary Tables

		Undiagnosed			Diagnosed		
		TR1	TR2	TR3	TR1	TR2	TR3
Undiagnosed	TR1		0.6231	0.1004	0.6629	0.1983	**0.01610**
	TR2			0.4891	0.3905	0.5535	0.07006
	TR3				0.06559	0.9877	0.1318
Diagnosed	TR1					0.1206	**0.01053**
	TR2						0.1937
	TR3						

Supplementary Table 1. Chi-square test results on distribution of sex. Pearson's Chi-square test shows pairwise comparison among groups of trajectories and diagnosis on distribution of sex (female, male). See Table 1 in main text for the numbers. The following table summarizes chi-square test results (p-values) and highlight significant outcomes in bold letter and green background.

		Undiagnosed			Diagnosed		
		TR1	TR2	TR3	TR1	TR2	TR3
Undiagnosed	TR1		**0.0004428**	**0.001546**	**< 0.0001**	**< 0.0001**	**< 0.0001**
	TR2			0.5951	**< 0.0001**	**< 0.0001**	**< 0.0001**
	TR3				**< 0.0001**	**< 0.0001**	**< 0.0001**
Diagnosed	TR1					0.8421	0.05336
	TR2						0.1296
	TR3						

Supplementary Table 2. Chi-square test results on distribution of HLA. Pearson’s Chi-square test shows pairwise comparison among groups of trajectories and diagnosis on distribution of HLA-DR status (DR3/X, DR4/X, DR3/4, DRX/X, Unknown). See Table 1 in main text for the numbers. The following table summarizes chi-square test results (p-values) and highlight significant outcomes in bold letter and green background.

Model Input					Model Output
Participant ID	GADA	IAA	IA-2A	Age	HMM State
Participant 1	0	0	0	0.5	3 (TR2-0)
Participant 1	0	1	0	1.0	4 (TR2-1)
Participant 1	0	1	0	1.6	4 (TR2-1)
Participant 1	0	1	1	3.3	5 (TR2-2)
Participant 1	1	1	1	4.7	6 (TR2-3)
Participant 1	1	1	1	6.2	6 (TR2-3)
Participant 1	1	0	1	7.0	7 (TR2-4)
……					
Participant 2	0	0	0	0.75	0 (TR1-0)
Participant 2	0	0	0	1.55	0 (TR1-0)
Participant 2	0	0	0	1.8	0 (TR1-0)
Participant 2	1	1	1	2.7	1 (TR1-1)
Participant 2	1	1	1	3.5	1 (TR1-1)
Participant 2	1	0	1	4.1	1 (TR1-1)
Participant 2	1	1	1	5.0	1 (TR1-1)
Participant 2	1	1	1	6.1	1 (TR1-1)
Participant 2	1	1	1	7.0	1 (TR1-1)
Participant 2	0	0	1	7.9	2 (TR1-2)
Participant 2	0	0	1	8.8	2 (TR1-2)
……					
Participant 3	0	0	0	1.9	8 (TR3-0)
Participant 3	0	0	0	3.6	8 (TR3-0)
Participant 3	1	0	0	6.3	9 (TR3-1)
Participant 3	1	0	0	7.6	9 (TR3-1)
Participant 3	1	0	1	8.1	10 (TR3-2)

Supplementary Table 3. Description of the input data table. To generate type 1 diabetes progression trajectories, we need four input data variables: GADA, IAA, IA-2A, and Age for a series of samples (visits) per participant. Then, the Hidden Markov Model is trained with the input data. As a side effect of the training, each visit is labelled with a latent state, as illustrated in the HMM State column.

Supplementary Discussion

Looking more closely at each trajectory, 215 of 256 children in TR1 entered TR1-0 at median age of 0.26 years old and stayed in the state for approximately 1.76 years. Of these, 46 children seroconverted and 11 were diagnosed at this initial state. In the next state, TR1-1, median age of first IAb appearance was 2.76 years for 244 children (204 from TR1-0 and 40 that started at this state). Median dwell time was 2.99 years, and it was the last state for 171 children. 145 children seroconverted and 167 were diagnosed at this state. The final state, TR1-02, includes 78 children, whose median age at first visit was 5.18 years old and whose median dwell time was 4.81 years.

In TR2, 199 out of 273 children entered TR2-00 at median age of 0.26 years and stayed in the state for approximately 1.24 years. No children seroconverted and only one child was diagnosed at this initial state. In the next state, TR2-01, median age of first IAb appearance was 1.49 years for 261 children (198 from TR2-00 and 63 that started at TR2-01). Of these, 205 children seroconverted and 83 were diagnosed at this state. The next state, TR2-02, included 185 children, whose median age at first visit was 2.99 years and with median dwell time of 1.27 years. Of these, 35 children seroconverted and 88 were diagnosed. In TR2-03, where all three IAbs were positive with a probability higher than 0.95, 101 children entered the state at median age of 3.50 years and stayed for a median of 4.21 years. Of these, 5 seroconverted and 64 were diagnosed. TR2-04, the last state of TR2, occurred much later at median age of 6.4 years than its preceding state. Here, no child seroconverted, and 37 children were diagnosed at the state.

In TR3, 94 out of 114 children started their trajectories at TR3-00 at median age of 0.25 years and stayed in the state for 2.8 years. Only two children seroconverted, and no child was diagnosed at the state. Then, the next state TR3-01 with GADA positive (0.98) includes 112 children (85 seroconverted; 41 were diagnosed) whose median age at first visit was 3.94 years with median dwell time of 2.73 years. The final state of TR3, TR3-02, started at median age of 6.28 years and had 5.03 years of dwell time for 73 children (16 seroconverted; 73 were diagnosed).

To examine state distribution among those who did not develop diabetes during the study periods, the visits of 1502 undiagnosed (UD) were assigned with labeled states and distributed into appropriate trajectories. In total, there were 483, 257, and 762 in TR1, TR2, and TR3, respectively. Of the undiagnosed, 628 (42%) transitioned into IAb positive states beyond initial states. More than half of them (N=365; 58%) belonged to TR3, while 63 (10%) children belonged to TR1, and 200 (32%) belonged to TR2. Mean ages of participants among the undiagnosed were consistently older than those of the diagnosed for all states.
